# The Digital Gender Gap in Teacher Education: The TPACK Framework for the 21st Century

**DOI:** 10.3390/ejihpe11040097

**Published:** 2021-10-25

**Authors:** Isabel María Gómez-Trigueros, Cristina Yáñez de Aldecoa

**Affiliations:** 1 Department of Specific Didactics, Faculty of Education, University of Alicante, 03690 Alicante, Spain; 2 Interdisciplinary Research Group in Education, University of Andorra, AD600 Sant Julià de Lòria, Andorra; cyanez@uda.ad

**Keywords:** digital gender gap, ICT, teacher education, TPACK, Teaching Digital Competence

## Abstract

The main goal of this research is to explore whether there are any differences by gender regarding the Digital Competence of Teachers (DCT), both in-training and in-service. Simultaneously, the specific goals of the research are to analyse which are the methodologies, including technological, that are being implemented in university classrooms and to evaluate possible new interventions to reduce the digital gender gap. This study is exploratory and descriptive. It relies upon three instruments that have been validated by experts: a questionnaire to collect teachers’ in-training perception, a second questionnaire to show in-service teachers’ perception regarding their knowledge of technologies, and a rubric to analyse in-service teachers’ self-perception regarding methodologies that employ technology. Over three academic years, data were collected from a sample of 914 trainee teachers and 194 professors from several Spanish universities. The results show that, concerning the teaching task, compared to men, the female participants have a very poor self-perception in terms of their Digital Teaching Competence, as well as a lower predisposition towards technologies. We conclude by emphasising the need to transform teaching methodologies in initial teacher education by means of the correct inclusion of ICT tools.

## 1. Introduction

Today’s Information and Knowledge Society (IKS) leads us to consider the need for significant changes in the production and organisation of work, in the media, in the way we conduct politics and identify the economic and social development of a country [1,2], and even in the way we relate to each other [3,4].

Both governments and educational administrations propose various actions, some of which have been embodied in laws, directives, and programmes, to enable citizens to enjoy access to and training *in* and *with* technologies, among other aspects. Certain social, economic, and technological conditions are essential to consolidate the knowledge economy and to contribute to the social and cultural development of countries, helping individuals to achieve their desired goals and resolve challenges in the professional employment and academic fields, which society sees as an essential condition in the values scale required in a global world.

UNESCO [5] presents itself as a promoter of the social dimension of technologies, recognising them as key features of the IKS. Several declarations [5] support the development of national policies and general plans on the use of Information and Knowledge Technologies (ICT) in education. The aim is to help governments to harness the potential of technologies in education systems with a view to achieving Sustainable Development Goal 4—Education by 2030. For its part, in order to ensure that all citizens have access to these ICT resources, the EU is setting up a series of programmes to monitor education and training in Europe. Thus, several programmes have been designed, such as *i-2010 from 2005 to 2009* and *Digital Agenda 2010* which, in the field of education, results in the e-Learning programme and, more recently, the Digital Education Action Plan 2018. The central goals of all of these programmes are, firstly, to provide appropriate educational hardware and software to achieve better employability through ICT and e-learning in teaching and training and, secondly, to promote the best possible use of innovative technology-based teaching and learning techniques.

Programmes such as the aforementioned ones—some of them, running until 2020, specifically aimed at increasing the amount of technological equipment in schools or training teachers and citizens in the manipulative use of ICT—are genuine examples of the administration’s concern regarding the new IKS scenario. However, these programmes do not incorporate the gender perspective in the design of their proposals. Accordingly, among the great challenges to be faced in this new context for the social construction of the 21st century, it is important to ensure that new elements of inequality between women and men should not be added or, better yet, that this new scenario should become a real chance to promote equality. Undoubtedly, the educational system should address these concerns.

Research into the digital gender gap is usually identified as a problem for women in ICT [4]. In this sense, studies on gender and technology have focused on quantitatively showing the scarce or non-existent presence of women in the field of study and the professionalisation of technologies [6,7]. However, more and more research tends to consider the digital gender gap a problem of enormous importance, along with the difficulties in accessing and using technologies and the development of basic computer skills [8,9]. We are facing a phenomenon linked to the under-representation of women in the strategic sectors of education, research, and employment linked to engineering and technologies in general [10] and, therefore, linked, also, to the male dominance in these sectors.

These distinctions regarding gender in the use of ICT represent and reproduce the pre-existing inequalities between women and men [6,11], so much so that, in academic reviews carried out by several authors [6,12], a large number of investigations on this subject have been collected, providing evidence of the scientific relevance of the topic. Therefore, the importance of research in this field is due to the social commitment to eliminate gender barriers that still persist today in many social spheres, among which are both the training of primary school and secondary school teachers.

Although the digital gender gap “is related to the male dominance of certain strategic areas such as education, research, and employment related to science, engineering, and ICT” [13] (p. 10), most of the research developed on this topic identifies it as a women’s problem with ICT [6,7]. In other words, the bulk of the studies “on gender and technology [have] focused on supplying documentary evidence of the numerical inferiority of women in the field of ICT studies, research, and occupation” [6] (p. 28).

However, more and more studies invite us to consider the digital gender gap as a problem of enormous importance, together with the “access to” and the “use of” ICT [8,14,15] or the development of basic computer skills. We are facing a phenomenon linked to the under-representation of women in the strategic sectors of education, research, and employment related to engineering and technologies in general [13,15,16] and, therefore, linked to male dominance in these sectors. Consequently, the study of the digital gender gap has gradually focused on the study of the explanatory factors of the under-representation of women in the world of ICT, with special emphasis on research on the disaffection of girls and young women by technologies and on the factors involved in their rejection and/or not considering careers in the field of technologies as an option [17,18].

These studies coincide in pointing out that ICTs are, both socially and culturally, defined as male and that the technological competency is understood as a male gender competency [6,19]. Studies such as these have led to a proliferation of programs and interventions, all focused on addressing these aspects to increase female students’ enrolment in such studies. Thus, we find a variety of recommendations, such as those that propose the creation of more favourable environments for girls and young women, to provide them with female examples in these fields. In this sense, there are proposals to work together with teachers in schools to carry out training and dissemination actions in computer activities [20], as well as to design some training in digital skills to promote non-differential manipulative use between women and men [21], among others.

Different recent research points out that these inequalities do not refer so much to the women’s presence (access to ICT) as to the women’s intervention in this field (management, use, attitude, existence of ICT content). Therefore, in this context, and from the existing literature, this emerging group research project proposes going further and developing studies related to the so-called third gender digital gap. This affects the diversity of actions that a user is able to take with ICTs, based on the quality and type of interaction established with the technologies [21].

In this interrelationship between active female and male users and technologies (and the Internet), female users acquire competitive advantages (information, knowledge and opportunities) compared to other individuals [22]. The fact is that if these competitive advantages are limited to the male population group, this will lead to female exclusion from digital citizenship. This is what has come to be called Beneficial and Advanced Uses of the Internet (UBAI) [23]. It is at this point where the importance of including new methodologies *with* and *in* ICT in teacher education to avoid UBAI gender differences between women and men should be emphasized. One of these T-L models that takes into account the appropriate inclusion and use of ICT in education is the Technological Pedagogical Content Knowledge (TPACK) framework.

At this point, we should emphasise the importance of including new methodologies *with* and *in* ICT in teacher education to avoid differences in use by gender. One of these teaching and learning (T-L) models, which considers the appropriate inclusion and use of ICT in education, is the TPACK framework. This model states that teachers must have pedagogical knowledge (PK), knowledge of the subject they teach (CK), and technological knowledge (TK) [14,24], known as deep teaching knowledge. In addition, the model stresses the importance of these three elements (CK, PK, and TK) interacting simultaneously in the T-L process. This is how a web of interrelationships is built. Teachers should be familiar with it and use it for the correct integration of ICT in their daily activities. The TPACK framework accepts that technology is here to stay. This new reality means that teachers must be trained in the use of technologies and in the skills to adapt to changes brought about by new software and hardware.

The TPACK framework provides an original point of view regarding the incorporation of ICT in the classroom, focusing not only on training in instrumental competencies but also on their interrelation with the didactic component. Accordingly, the competencies that should be emphasised in initial teacher education are cognitive, methodological, attitudinal, and emotional. Their mastery and understanding will allow the correct use of technologies in teaching [4]. Likewise, this model shows itself capable of resolving conflicts in initial teacher education, helping teachers to develop changes in procedures concerning technologies. To this end, the model proposes a reflective action when approaching educational work, helping to meditate the teacher’s training as it enables him/her to participate in self-knowledge and self-development processes in didactic practice [9]. Therefore, the focus is on the process of T-L with technologies and not on how to introduce ICT to teach specific disciplinary content.

This research addresses the complex issue of educating and training teachers in digital competency training with regard to gender. The approach will make it possible to verify sexist features in the access, use, and promotion of technologies; to analyse didactic methodologies and teaching and learning models (T-L) proposed in primary school teaching degree and master’s degree curricula in regards to ICT training; to evaluate whether equal access of women and men to technologies is assured in digital teacher education in initial training; and, finally, to promote an appropriate, non-sexist technological education to train teachers in the better manipulation and didactic use of digital tools for teaching citizenship. After analysing the existing situation regarding the digital gender gap, the TPACK framework is recommended. The aim is to present a teaching and learning model for teachers both in-training and in-service, a model that takes technologies into account while also seeking to reduce the gender gap in ICT use.

## 2. Materials and Methods

This research took place within the context of the Education faculties of several Spanish universities, within a more ambitious research project on the digital gender gap that encompasses other Spanish and international universities. The research was developed over three different academic years. In this sense, the proposed methodology is based on analysis of initial teacher education from two different but complementary points of view. Firstly, observation and visualisation of the existence or not of the digital gender gap in the training of future teachers, in order to quantify the current situation, was conducted. To this end, a study of the current situation in the classroom with regard to the inclusion of technologies was carried out, considering students’ perception of their training in Digital Competence (DC). Secondly, the methodologies *with* and *in* gender-inclusive technologies that are currently implemented in this initial training were analysed. The research takes on an exploratory and descriptive nature with the purpose of examining, describing, and interpreting how initial teacher education in Teachers’ Digital Competence (TDC) is being carried out from a gender perspective. Thirdly, in-service teachers’ self-perception in relation to the importance and use of ICT resources in their usual tasks in future teacher training was collected.

### 2.1. Participants

With regard to the participants, a representative sample of 914 students (primary school teaching degree and master’s degree students) (Table 1) was selected out of a total population of 1025, which represents 68% of the total number of potential individuals to be studied. This non-probabilistic sampling of students (*n* = 803) available (in relation to the indicated subjects) is adequate and enables experimental research of the factorial type, between groups, to be conducted.

Specifically, the responses regarding the perception of 621 women (68.3%) and 293 men (31.7%) were analysed. Their distribution, by studies, is as follows:

Moreover, the responses of 194 teachers from these same universities were analysed over three academic years (2018–2019, 2019–2020, and 2020–2021). As in the case of the students, this is a non-probabilistic, available sampling (in relation to the subjects indicated and who voluntarily agreed to participate) of teachers of the degrees of primary school teaching and master’s degree in teaching (*n* = 387). This sample is adequate and allows experimental research of a factorial type between groups to be conducted.

Specifically, the responses of 111 women (57.2%) and 83 men (42.8%) were analysed (Table 2). Their distribution, by studies, is as follows:

### 2.2. Research Instruments

Various resources were developed as research instruments: firstly, a questionnaire for students (Questionnaire 1), on a Likert scale, consisting of 37 items; secondly, a four-level rubric for teachers on the methodology implemented in the classroom; and thirdly, a questionnaire for teachers (Questionnaire 2), on a Likert scale, consisting of 37 items. (For further information on the analytical instruments employed, please contact the authors.)

The first instrument (Questionnaire 1) tackles, in a concrete manner, aspects related to the sociodemographic characteristics of the sample (items 1–2), as well as the academic year and studies they are taking (items 3–5). It is divided into seven blocks: (1) the perception of their own technological knowledge (items 6–9); (2) the perception of the subject (items 10–13); (3) pedagogical knowledge (items 14–20); (4) pedagogical and disciplinary knowledge of the content (items 21–23); (5) knowledge of technologies for the transmission of content (items 24–26); (6) knowledge of technology and pedagogy (items 27–35); and (7) perception of the methodologies implemented with technology in their training, their training for its use, and the T-L models to carry out a correct inclusion of ICT in the classroom (items 36–37).

All instruments were correctly validated by experts from Spanish and foreign public universities, specialists in the subject, following the method of the Panel of National and International Experts. This expert panel method involves consultation of scholars who have great knowledge of the subject of the research (all curricular areas, as described in current Spanish regulations), as well as of the context in which the study is intended to be implemented (primary schools). This panel was made up of specialists (more than 10 members) from different backgrounds (universities of Burgos, Elche, Murcia, Salamanca, Albacete, Balearic Islands Cuba, Ecuador, and Portugal), guaranteeing complementary and diverse points of view. They lent their knowledge on specific didactics and methodologies using technology, TPACK, and, also, the gender gap to help interpret not only the proposed intervention but also the questionnaires drawn up. The appropriate composition of the panel, as well as the members’ level of knowledge and diverse backgrounds, were all elements that helped to guarantee the success of the prospective activity [25,26].

In order to ascertain the reliability of the questionnaires, Cronbach’s alpha coefficient was calculated [27]. The results obtained (α = 0.931) confirm the existence of a high and adequate internal consistency of the tool for the proposed study [28]. Similarly, Pearson’s Chi-Square index was administered, producing results of *p*-value < 1 = Sig. 0.001 [29], confirming not only the high correlation of the questions posed but also the validity of the items and the structure of the instrument implemented.

It should be noted that the TPACK framework, selected as a structuring element of our research, studies precisely the integration of technology in education. As previously stated in the introduction section of this article, this model is based on the combination of three variables wherein each teacher must be trained: technological knowledge (TK), pedagogical knowledge (PK), and content knowledge (CK). More and more universities and educational centers are implementing technological tools to incorporate them into their students’ learning. This process requires teachers to adapt to technological change and to work on these skills. Thus, in combination with their pedagogical, disciplinary, and content knowledge, an educational environment that effectively integrates Information and Communication Technologies (ICT) will be achieved.

Through seven dimensions, the TPACK framework organizes the relationship between content, pedagogy, and technologies. For this research, we have built our tool starting from those dimensions pre-designed by the framework itself. Thus, we have adapted the questionnaires of other researchers from the TPACK framework [5,30,31,32]. In this sense, it has not been considered necessary to carry out, for example, a factor analysis of grouping of variables, reduction in variables, or elimination of certain questions to shorten the questionnaires, since the dimensions described by the model are the ones that have been used: TCK or technological knowledge of the content; TPK or pedagogical technological knowledge; and TPACK or Pedagogical Technological Knowledge of Content. It also has not been necessary to carry out a cluster analysis, in this case, grouping variables, since, as indicated, the dimensions described by the model have been used and adapted. Regarding the verification of the scale or questionnaire, it should be noted that the Cronbach’s Alpha process has been used.

Likewise, in order to analyse the methodologies implemented by the teaching staff at the different universities in this study, an evaluation was carried out using a rubric (second instrument of analysis, in which four dimensions of analysis were collected). The first dimension is related to the didactics, the curriculum, and the methodology used by the participating teachers; the second dimension is related to the planning, organisation, and management of digital technological resources; the third dimension concerns the relational aspect, ethics, and security; and the fourth dimension is related to professionalism in the use of technological tools (Chart 1) based on the adaptation of the European Commission’s DIGCOMP [33] and Lázaro-Cantabrana et al. [34].

**Chart 1:** Self-assessment rubric of ICT methodologies for participating teachers.

Dimension	Descriptive
**1**	1.1. Teaching, planning, and Teachers’ Digital Competence.
	1.2. Use and consideration of digital technologies as tools for teaching and learning (T-L).
	1.3. Information processing and knowledge creation with ICT.
	1.4. Methodological line of the curricular design of the subject.
**2**	2.1. Learning environments with ICT in the classroom.
	2.2. Management of digital technologies and implementation in relation to the selection, use and combination of ICT in L-T.
	2.3. Digital technological infrastructures in relation to the inclusion, responsible use, and management of ICT in the educational processes.
**3**	3.1. Ethical and safe use of resources.
	3.2. Inclusive digital, non-sexist implementation of ICT in the classroom.
	3.3. Communication, dissemination, and knowledge transfer based on the technologies and resources used.
	3.4. Creation and use of digital content for the entire educational community.
**4**	4.1. Free access to information, production and dissemination of educational material with open, accessible licences.
	4.2. Leadership in the use of digital technologies with students.
	4.3. Personal Learning Environment (PLE).

Source: Prepared by the research team.

An expert focus group from different national and international universities was set up to adapt the rubric or assessment matrix. These were experts in methodologies, educational technology inclusion, and didactics. Professional profile, experience, and diversity in terms of institutional representativeness were the criteria used to form the group.

The validation process was carried out in two different phases. The first was remote, based on providing the evaluators, prior to the synchronous session via Google Meet, with the existing and already published papers [22,23,33]. This first stage focused on analysing the dimensions and associated indicators on the basis of three criteria: clarity, relevance, and priority of the indicators, grouped by dimension. In order to share individual evaluations and achieve a necessary consensus in decisions to carry out the adaptation of the rubric, the discussion group met and was moderated by one of the researchers involved in this paper.

Regarding the conceptualisation of terms, it was decided to adapt several, such as students, programming, curricula, initial teacher education, teachers, etc. These were more appropriate in the Spanish educational context, the setting wherein the study was implemented.

Regarding relevance, it was agreed that all the indicators were appropriate and that they also correctly measured the dimension in which they were found.

Once the individual contributions of the group members had been assessed, the order of the indicators in each dimension was established. This means that, when using the rubric as an instrument for assessment, the order of the indicators that are considered more or less fundamental as components of the different dimensions of the teachers’ use of methodologies *in* and *with* technologies can be taken into account.

The third instrument (Questionnaire 2) analyses, specifically, those aspects related to the sociodemographic characteristics of the sample (items 1–2), the academic year they are teaching in, and the number of years of university teaching (items 3–5). It is divided into seven blocks: (1) the perception of their own technological knowledge (items 6–9); (2) the perception of the subject (items 10–13); (3) pedagogical knowledge (items 14–20); (4) pedagogical and disciplinary knowledge of the content (items 21–23); (5) knowledge of technologies for the transmission of content (items 24–26); (6) knowledge of technology and pedagogy (items 27–35); and (7) perception of the methodologies implemented with technology in their teaching and training and for their use in the T-L models to ensure the correct inclusion of ICT in the classroom (items 36–37).

As in the case of Questionnaire 1, the instrument was duly validated by experts specialising in the subject, both from Spanish and foreign public universities, using the National and International Expert Panel Method. The reliability of Questionnaire 2 was also confirmed by calculating Cronbach’s alpha coefficient [27]. The results obtained (α = 0.902) confirm the existence of a high and adequate internal consistency of the tool for the proposed study [28]. Likewise, Pearson’s Chi-Square index was calculated, with results of *p*-value < 1 = Sig. 0.001 [29], indicating the high correlation of the questions posed and the validity of the items and the structure of the instrument implemented.

### 2.3. Method Design

Regarding procedure, the questionnaires were distributed by e-mail using the free Google Forms application in different academic years (2018–2019, 2019–2020, and 2020–2021). The participants were informed about the aim of the research, as well as the confidentiality with which their replies would be treated.

Once the information had been collected from the groups (students and teachers), the quantitative data were analysed using the SPSS v.25 statistical package. Taking into account the proposed objectives, different tests were carried out. In the initial phase, the internal consistency of the questionnaire was assessed and the main descriptive statistics of the set of quantitative responses were obtained (mean = *M* and standard deviation = *SD*). After this, in a second phase, an exploratory analysis of the descriptive test statistics was carried out according to the dimensions that make up the instrument. In a third phase, different statistics were applied (Student’s *t*-test and ANOVA of one factor), enabling us to verify in detail the existence of significant differences by sex.

In relation to the rubric, it should be pointed out that it was exhaustively evaluated through a self-evaluation process by the participating teachers. Its distribution was made using Google Forms. The teachers responded anonymously to each item, indicating their self-perception. With regard to the responses, only two options were offered: *presence* or *absence* of such actions/issues in their work with trainee teachers (For further information on the rubric employed, please contact the authors.)

## 3. Results

### 3.1. Descriptive Analysis

Regarding the descriptive analysis of the participants’ scores, presented here are those from block 5 (items 24–26), which refer to knowledge of technologies for content transmission; block 6 (27–35), focusing on knowledge of technology and pedagogy; and block 7 (items 36–37), relating to the perception of the implemented methodologies with technology in their training, as well as the ability to use them, and T-L models for the correct inclusion of ICT in the classroom. Comparison of the descriptive statistics results (*M*; *SD*) for each of the blocks (Table 3) according to gender shows, in general terms, that the perception of the male participants, in all the analysed dimensions, is very positive, with means close to a value of 5 (*Totally agree*), among both students and teachers and with deviations that are indicative of a high consensus among the replies received (*SD* ≤ 0.573).

In questions referring to knowledge of technology for teaching (TK) (items 24–26), the most significant differences are presented between the responses provided by women (*M* ≤ 2.33; *SD* ≤ 0.459) and those provided by men (*M* ≥ 4.53; *DT* ≤ 0.553). These values show the different perceptions, by sex, regarding knowledge of technological resources and training in Learning and Knowledge Technologies (LKT) for teaching by students and teachers.

The data on responses regarding knowledge of technology and pedagogy (PTK) (items 27–35) show a negative perception among women, notably in those questions related to their ability to provide others with leadership; to help others coordinate the use of content, technologies, and teaching strategies (item 34); and to choose certain technologies to enhance the content of a lesson (item 35). In both questions, the women’s responses yield a value of *M* ≤ 2.25, relative to the Likert questionnaire response option “*Disagree.*” In contrast, men’s response options are close to value 5, “*Fully agree*” (*M* ≥ 4.88).

Regarding the perception of the implemented methodologies with technology in their training for T-L (TPACK) (items 36–37), the values also show remarkable differences by gender. Thus, the results obtained for women (*M* ≤ 2.45) suggest a negative perception regarding their ability and training in this type of T-L model. On the other hand, this same dimension yields positive values for men, approaching the response option “*Fully agree*” (*M* ≥ 4.58). These values indicate the different perception regarding their skills and training for the proper use and implementation of T-L models with technologies between male and female teachers in-training.

### 3.2. Comparative Analysis by Gender; ANOVA and Students’ t-Tests

After the descriptive analysis of the items, a comparison of means was carried out through a *t*-test for independent samples, as well as a univariate analysis of variance using the one-way ANOVA analysis technique (Table 4). The purpose is to compare and assess the existence or not of significant differences by gender among students and teachers who make up the sample in relation to the analysed blocks.

According to the findings, the existence of significant differences among the groups studied (students, teachers in-training, and professors who train these students) in the variables studied can be confirmed.

The confidence interval limits for the difference show that, for the variables, in both groups of studies, the value 0 is not included between the confidence interval limits. This is indicative that, in these items, the hypothesis of equality of means can be rejected, as is already confirmed by the results of the *t*-test for these variables (see Table 4).

The results from the analysis of variance show the existence of significant differences between women and men in their assessment of the three dimensions (TCK, TPK, and TPACK) in the groups analysed (students and teachers) (*p* < 0.01). Thus, in the variables related to knowledge of disciplinary and technological content (TCK dimension) (item 24 *p* = 0.001; item 25 *p* = 0.003; item 26 *p* = 0.005), male participants attach greater importance to this subject than do female participants, among both students and teachers. A similar result is found regarding the variables of the TPK dimension (item 27 to item 35 *p* ≤ 0.008), where male participants value it very positively (*M* ≥ 4.69), with a mean value close to 5 (*Fully agree*) compared to a value of 4 (*M* ≤ 4.16) (*Agree*) with women. Additionally, it is the male participants who consider as relevant skills and abilities the symbiosis between content, the correct use of technologies, and pedagogical knowledge when working in the classroom (TPACK dimension) (item 36 *p* = 0.004; item 37 *p* = 0.006). In general, for these variables, significant differences are found between participants (women and men) with higher mean values, in the variables, among men (men = *M* ≥ 4.79; women = *M* ≤ 4.03).

In this research, it was decided that an effect size analysis based on differences between sexes using Cohen’s d tests would be carried out. The results of the *t*-test were also taken into consideration. In both cases, a medium–high value effect size (>0.8) is found [35,36]. This shows the magnitude of the result found, which allows us to offer an estimate of the scope of our investigation. These results lead us to continue investigating this topic. (See Table 4.)

In this sense, the size of the eta squared effect for ANOVA yields a medium–high value (≥0.9), indicating that the effect size by gender is large. These results coincide with the *p*-values shown in the output of the ANOVA table (Table 4).

### 3.3. Analysis of the Rubric on Self-perception of the Participating Teachers’ Methodologies

Together with the statistical analysis conducted on the responses to the questionnaires (Questionnaire 1 and Questionnaire 2), results were also extracted from the rubric that had been distributed to the teaching staff participating in the research.

**Figure 1:**
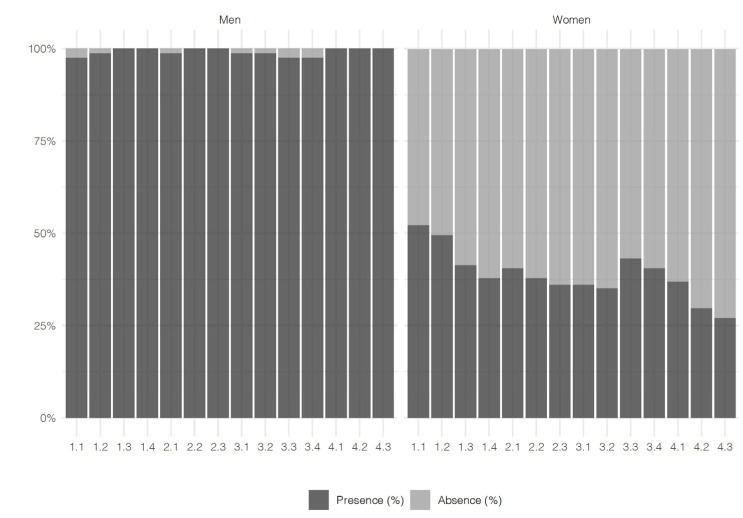
Results of the self-perception rubric of absence–presence of the items. Source: Prepared by the research team.

In the four dimensions studied (Chart 1), the self-perception of male teachers is higher than that of female teachers (Table 5, Figure 1). A more detailed analysis shows that, in Dimension 1, relative to the didactics, the curriculum, and the methodology used by the participating teachers, male teachers respond with a frequency of ≥97.5% in the four requested subsections of the option, that there is a “*presence*” of such consideration in their classroom proposals: 1.1. Teaching planning and Teachers’ Digital Competence (97.5%); 1.2. Use and consideration of digital technologies as T-L tools (98.7%); 1.3. Information processing and co-creation of knowledge with ICT (100%); and 1.4. Methodological line of the curricular design of the subject (100%). On the contrary, female teachers indicate a lower presence of such considerations in their classrooms. Thus, in Dimension 1.1, only 52.2% state that they consciously take this dimension into account in their teaching organisation; in Dimension 1.2, 49.5% take into account technologies for teaching; in Dimension 1.3, 41.4% of female teachers carry out content creation with technologies; and in Dimension 1.4, only 37.8% engage in activities aimed at developing teaching and learning according to the methodological guidelines and resources provided at the institution and incorporate, in their teaching work, Digital Competence in meaningful activities, which entails the use of digital technologies to build and share knowledge.

Something similar occurs when we analyse the results of Dimension 2, related to the planning, organisation, and management of digital technological spaces and resources. Here again, the male teachers are the ones who report a greater “*presence*” in their classrooms, as well as in their methodologies, of the different subsections studied (always higher than a frequency response of 98.7%) and with a 100% affirmative response in item 2.2: Management of digital technologies and applications in relation to the selection, use, and combination of ICT in T-L, and item 2.3: Digital technological infrastructures in relation to the inclusion, responsible use. and management of ICT in-training processes. These values show that male teachers attach greater importance to technological resources, as well as to future teachers’ DC training.

If we analyse the answers given by the female teacher participants, we note a lower presence of such technological elements in the organisation of their teaching and in the development of their classes. In this sense, in Section 2.1, related to learning environments, only 40.5% recognise the “*presence*” of digital classroom technologies such as PDI, fixed and mobile devices, according to each E-Learning situation. This indicates a low level of adoption of E-Learning activities compared to the digital technologies available at the academic institution. Something similar occurs with Section 2.2, on the management of digital technologies and applications, where only 37.8% respond with the option “*presence of interventions*” in their teaching practice, on the use of different digital technologies according to their potential, and reflexively analysing the performance of students based on their use. In relation to Section 2.3, related to digital technological infrastructures and their responsible use, only 36.0% of the female teachers highlight this as a “*presence*” in their regular work.

When observing the responses for Dimension 3, which includes the teachers’ self-perception of ethics, safety, and the use of technologies and digital content in teaching, a higher amount of “*presence*” of such considerations is found among male teachers (≥97.5% of “*presence*” responses) than among female teachers (≤43.2% of “*presence*” responses). More specifically, a greater number of “*absence*” responses from female teachers is significant for all the subsections studied: 3.1: Ethical and safe use of resources = 63.9%; 3.2: Inclusive digital, non-sexist implementation of ICT in the classroom = 64.8%; 3.3: Communication, dissemination, and transfer of knowledge from the technologies and resources used = 56.7%; 3.4: Creation and use of digital content for the entire educational community = 59.4%. This is compared to the low frequency of the “*absence*” response among male teachers (3.1. = 1.3%; 3.2. = 1.3%; 3.3 = 2.5%; 3.4. = 2.5%). Such values corroborate the different importance that male versus female teachers give to promoting access to and use of digital technologies by all students, with the aim of compensating for inequalities or the consideration of being a model in their classrooms in the ethical use of digital technologies during activities with students.

Finally, Dimension 4, which is related to professionalism in the use of technological tools, is precisely where the most differences by gender are detected (Figure 1). Thus, in the three subsections of this dimension (4.1. Free access to information, creation, and dissemination of didactic material with open, accessible licences; 4.2. Leadership in the use of digital technologies with students; 4.3. Personal learning environment), male teachers respond with 100% to the option “*presence*”. In contrast, we have the responses of female teachers, which do not reach 37% in any of the subsections of this dimension, the “*presence*” option (4.1. = 36.9%; 4.2. = 29.7%; 4.3. = 27.0%). Such responses are significant to the “*absence*”, on the part of female teachers in their teaching work, of the following: the development of open didactic materials; sharing such teaching materials online following a standard to facilitate search and accessibility; coordinating the use of digital technologies either at the college or the university; lifelong training through activities related to digital technologies recognised by their educational administration; and collaborating with or advising teachers in their faculty or university with regard to the creation of Personal Learning Environments (PLE).

## 4. Discussion

Recent studies show that the first digital gender gap is gradually disappearing, with a tendency to equalise the number of ICT users of both sexes [16,37,38]. However, the second digital gender gap in today’s society [13,39], which is determined by gender differences in technological skills, is still to be overcome [40].

In the current context, the problem is considered to be the result of certain social practices that contribute to a low female preference for technology, leading to male dominance in those working fields where new technologies are being developed [16,40].

Disparities in the digital gender gap between EU countries are observed. The 2020 Digital Economy and Society Index (DESI), which monitors Europe’s overall digital performance and tracks the progress of EU countries with respect to their digital competitiveness, has five indicators for digital performance: connectivity, human capital, Internet use, digital technology integration, and digital public services. This index ranks Finland in the first place in the EU Member States list. This is due to public investment in training and technological resources and in equitable and inclusive training [41]. On the contrary, at the opposite extreme, are countries such as Bulgaria, Slovenia, and Romania, which have a low rate of connectivity and training in digital skills for women.

In the case of Spain, the actions that have been implemented place it in the EU average. However, the data from this research shed light on how much remains to be done, hence this research on the need to train teachers in digital skills.

While literature on the study of the digital gender gap and the importance of initial teacher education in the use and implementation of technologies in teaching is scarce, it is important to underline that most such studies have focused on showing the differentiated use of these resources among women and men but without addressing the educational field [42]. This work, however, delves into issues related to the digital gender gap based on the perception that both teachers-in-training and professors of the Faculties of Education report as regards their didactic knowledge, their training in the use of technologies, and their frequency of use of such resources. It should be noted that the correct use and inclusion of ICT in classrooms depends on the training received. Therefore, it is necessary to address how it is carried out and what methodologies are implemented [43].

It is necessary to go back to the 1990s to find when the term “digital gap” emerged [44] (p. 67), differentiating between those who “have or do not have Internet, network and computer access.” The term “digital gap” arose from analysis of the incidence of ICT in the social structure. This situation determines the position of individuals and communities in the world and reinforces the existing differences between countries. At the same time, it generates an enormous inequality between those fully integrated into technological development and those who remain in the margins.

Several studies base the existence of the gender digital gap on a low participation of women (computer scientists, programmers, and telecommunications engineers) in the design and programming of software and hardware, as well as “in (…) the possibilities of participation in the same design and technological development” [45]. Other research draws attention to gender differences in the use of ICT in education [11,46,47] and provides evidence that there is a greater dominance of men compared to women in the management of these technologies. This research also suggests that men have a more positive attitude to ICTs, while women present a negative view towards them [18,48,49]. Such studies demonstrate a stereotypical attitudinal coping by women and men towards social networks [48].

The results obtained from this study show that future female teachers participating in the study have a poor perception of their Digital Competence (DC) and, also, of their Teaching Digital Competence (TDC). On the contrary, the values obtained in the responses given by the male participants were positive and, in certain cases, very positive. Such results on the negative perception of women regarding their training in technological resources converge with the research of other authors [12,50].

This study also confirms that in the self-perception of active female teachers there is a scarce presence of those dimensions recognised as inherent to the TDC of university teaching professors [14,24]. In this regard, recent studies [35,51] show low female participation and little interest in their training and improvement in the use of certain technological devices [52]. These results match with those from this case study wherein the differences between male and female teachers highlight the lack of importance attached to certain aspects that are considered essential to carry out the correct training in DC for future teachers. Specifically, among female teachers, absences are detected regarding the ability to select, evaluate, and use the most appropriate digital technologies for the development of the teaching task, inside and outside the classroom. Likewise, among the female teachers, throughout their work in the classroom, the absence of the implementation of a specific responsible use of the organisation and management of technologies and digital spaces at their university centres was noted. Such considerations pose limitations not only for professional development but also for the growth of those individuals with low qualifications in Digital Competencies [53]. This will have a negative impact on the training of future teachers, thus reproducing the current structures and negative self-perception regarding technologies by women.

Similarly, other authors [54,55] have identified several typologies of ICT models used in the classroom: on the one hand, a weak didactic integration and, on the other, an intense didactic integration. In addition, these authors found that teachers who use digital technologies to communicate and build knowledge (regular ICT users) perceive that they have adequate digital training and are also the ones who perform a correct didactic integration of these in the classroom. From the analysis carried out in this study, it appears that those teachers who carry out a responsible, legal, and safe use of technological resources also carry out a constant improvement of their professional practice that reflects their recognition of a digitised society that must be trained in TDC. These results are consistent with the studies mentioned above, as well as with the statements of Gil-Juárez et al. [42] and Acosta and Pedraza [53]. These analyse the inequalities in the use of and access to technologies by gender, based on knowledge of ICT, skills to access them, and, finally, skills for job performance in environments related to these resources. They make it clear that the gaps which have opened regarding access to and use of ICT, as well as the low participation of women in the implementation of technologies, confirm a low motivation and a negative social perception of women towards such resources in the work and training fields.

Likewise, there are differences between male and female teachers in relation to the training they receive for the correct inclusion of ICT in the classroom. In this sense, women consider that they do not have adequate T-L training in methodologies that use technologies. For their part, however, men do perceive that they have received adequate training in these educational models. Such results, which are consistent with similar works [15,54,55], highlight the enormous differences that both sexes have in the training received, which affects the way they approach their activity as teachers. These statements are corroborated in the current study, wherein male teachers show a positive self-perception about their use of methodologies with technologies compared to female self-perception about these same aspects. The reasons behind this different perception may lie in competency, sociocultural factors (traditionally, men are enabled to acquire greater digital skills), or simply attitudinal factors on the part of both sexes.

All these data come to confirm the enormous task that is still pending in the initial training of teachers, evidencing substantial differences in the perception of digital teacher education among women and men. In addition, the data highlight the need to influence the implementation of T-L models with technology, permitting the adequate preparation of future teachers in the use of such tools for their teaching activity, to mitigate the digital gender gap that continues to exist [5,12,54]. The results of this research lead us to recommend the creation of more conducive contexts for women through both the transmission of female models in the field of technology and the promotion of training actions aimed at improving teaching–learning processes supported by the new technologies among university professors [17,20,56,57]. The importance of the predominant methodological models and how they are implemented in classrooms should also be taken into account.

## 5. Conclusions

It should be noted that the differences between male and female teachers in relation to the correct use of technologies highlight their lack of training aimed at enabling them to deliver correct training in DC. More specifically, the results detect, among female teachers, a lack of ability to select, assess, and use the most appropriate digital technologies for the teaching task inside and outside the classroom. Indeed, these findings confirm the absence, among teachers of both sexes, of the implementation throughout their work, both in the classroom and in digital spaces at universities, of a responsible use aimed at organising and managing technologies.

## Figures and Tables

**Table 1:** Distribution by studies and gender of the participating students.

		Degree in Primary School Teaching	Master’s Degree in Teaching	Total
	**Women**	513	108	621
	**Men**	174	119	293
**Total**		687	227	914

Source: Prepared by the research team.

**Table 2:** Distribution by studies and gender of the participating teachers.

		Degree in Primary School Teaching	Master’s Degree in Teaching	Total
	**Women**	71	40	111
	**Men**	52	31	83
**Total**		123	71	194

Source: Prepared by the research team.

**Table 3:** Descriptive results (*M*; *SD*) of the items.

Item	Students			Professors				
	Sex							
	Women		Men	Women			Men	
	*M* ^1^	*SD* ^2^	*M*	*SD*	*M*	*SD*	*M*	*SD*
**Technological Content Knowledge (TCK)**								
Item 24	2.11	0.454	4.33	0.532	2.15	0.444	4.26	0.553
Item 25	2.21	0.432	4.58	0.502	2.21	0.459	4.22	0.543
Item 26	2.33	0.458	4.40	0.518	2.15	0.452	4.50	0.541
**Technological Pedagogical Knowledge (TPK)**								
Item 27	3.25	0.511	4.45	0.562	3.17	0.729	4.33	0.514
Item 28	3.64	0.559	4.78	0.556	3.65	0.788	4.39	0.526
Item 29	3.52	0.610	4.59	0.542	3.12	0.749	4.33	0.525
Item 30	3.24	0.605	4.56	0.568	3.15	0.715	4.41	0.571
Item 31	3.19	0.538	4.87	0.549	3.16	0.781	4.40	0.565
Item 32	3.42	0.584	4.71	0.556	3.21	0.797	4.50	0.559
Item 33	3.14	0.617	4.53	0.523	3.23	0.738	4.55	0.562
Item 34	2.21	0.581	4.84	0.527	2.25	0.775	4.88	0.519
Item 35	2.09	0.594	4.98	0.546	3.23	0.785	4.39	0.520
**Methodologies implemented with technology in their training for Teaching and Learning (T-L) (TPACK)**								
Item 36	2.43	0.629	4.55	0.548	2.42	0.765	4.49	0.565
Item 37	2.45	0.598	4.47	0.556	2.32	0.759	4.58	0.559

^1^ *M* = Mean; ^2^ *SD* = Standard or Typical Deviation. Source: Prepared by the research team.

**Table 4:** The *t*-test for independent samples and single-factor ANOVA analysis of variance.

Dimensions	Item	Analysed Group		*t* Student’s test		ANOVA	
		Women	Men	*t*	*p*	*F*	*p*
**TCK** **Dimension**	Item 24	3.26	4.01	−4.454	0.000 **	109.283	0.001 **
	Item 25	3.56	4.11	−1.547	0.000 **	30.771	0.003 *
	Item 26	3.72	4.07	2.752	0.006 *	7.573	0.005 *
**TPK** **Dimension**	Item 27	4.08	4.85	−5.326	0.000 *	28.363	0.002 *
	Item 28	4.09	4.88	−2.205	0.028 *	4.862	0.008 *
	Item 29	4.11	4.87	−3.674	0.000 *	13.498	0.002 *
	Item 30	4.01	4.69	−3.827	0.000 *	14.645	0.004 *
	Item 31	4.07	4.71	−2.961	0.003 *	8.767	0.007 *
	Item 32	4.09	4.73	−2.864	0.020 *	8.202	0.004 *
	Item 33	4.05	4.67	1.991	0.045	3.963	0.001 *
	Item 34	4.08	4.69	−3.140	0.049	9.859	0.002 *
	Item 35	4.16	4.71	−3.750	0.054	14.060	0.001 *
**TPACK** **Dimension**	Item 36	4.03	4.89	−2.876	0.011 *	8.269	0.004 *
	Item 37	4.01	4.79	0.139	0.012 *	0.019	0.006 *

According to the Levene statistic, equal variances for all outcomes (*p* > 0.05), * *p* < 0.05; ** *p* < 0.01. Source: Prepared by the research team.

**Table 5:** Quantitative results of responses to the rubric instrument.

		MEN				WOMEN			
		Presence	%	Absence	%	Presence	%	Absence	%
**Dimension 1**	**1.1**	81	97.5%	2	2.5%	58	52.2%	53	47.7%
	**1.2**	82	98.7%	1	1.35	55	49.5%	56	50.4%
	**1.3**	83	100%	0	0	46	41.4%	65	58.5%
	**1.4**	83	100%	0	0	42	37.8%	69	62.1%
**Dimension 2**	**2.1**	82	98.7%	1	1.3%	45	40.5%	66	59.4%
	**2.2**	83	100%	0	0	42	37.8%	69	62.1%
	**2.3**	83	100%	0	0	40	36.0%	71	63.9%
**Dimension 3**	**3.1**	82	98.7%	1	1.3%	40	36.0%	71	63.9%
	**3.2**	82	98.7%	1	1.3%	39	35.1%	72	64.8%
	**3.3**	81	97.5%	2	2.5%	48	43.2%	63	56.7%
	**3.4**	81	97.5%	2	2.5%	45	40.5%	66	59.4%
**Dimension 4**	**4.1**	83	100%	0	0	41	36.9%	70	63.0%
	**4.2**	83	100%	0	0	33	29.7%	78	70.2%
	**4.3**	83	100%	0	0	30	27.0%	81	72.9%

Source: Prepared by the research team.

## Data Availability

The data presented in this study are available upon request from the corresponding author. The data are not publicly available due to their format and length (e.g., completed questionnaires, database, etc.).
